# Prevalence and prognosis of molecularly defined familial hypercholesterolemia in patients with acute coronary syndrome

**DOI:** 10.3389/fcvm.2022.921803

**Published:** 2022-07-27

**Authors:** Cheng Wang, Puliang Yu, Lizhi Hu, Minglu Liang, Yi Mao, Qiutang Zeng, Xiang Wang, Kai Huang, Jin Yan, Li Xie, Fengxiao Zhang, Feng Zhu

**Affiliations:** ^1^Department of Cardiology, Tongji Medical College, Union Hospital, Huazhong University of Science and Technology, Wuhan, China; ^2^Clinic Center of Human Gene Research, Tongji Medical College, Union Hospital, Huazhong University of Science and Technology, Wuhan, China; ^3^Wuhan University of Science and Technology, Wuhan, China; ^4^Department of Clinical Laboratory, Tongji Medical College, Union Hospital, Huazhong University of Science and Technology, Wuhan, China; ^5^Clinical Research Institute, Shanghai Jiao Tong University School of Medicine, Shanghai, China

**Keywords:** acute coronary syndrome, cardiovascular events, familial hypercholesterolemia, low-density lipoprotein cholesterol, lipid

## Abstract

**Background:**

Familial hypercholesterolemia (FH) can elevate serum low-density lipoprotein cholesterol (LDL-C) levels, which can promote the progression of acute coronary syndrome (ACS). However, the effect of FH on the prognosis of ACS remains unclear.

**Methods:**

In this prospective cohort study, 223 patients with ACS having LDL-C ≥ 135.3 mg/dL (3.5 mmol/L) were enrolled and screened for FH using a multiple-gene FH panel. The diagnosis of FH was defined according to the ACMG/AMP criteria as carrying pathogenic or likely pathogenic variants. The clinical features of FH and the relationship of FH to the average 16.6-month risk of cardiovascular events (CVEs) were assessed.

**Results:**

The prevalence of molecularly defined FH in enrolled patients was 26.9%, and coronary artery lesions were more severe in patients with FH than in those without (Gensini score 66.0 vs. 28.0, respectively; *P* < 0.001). After lipid lowering, patients with FH still had significantly higher LDL-C levels at their last visit (73.5 ± 25.9 mg/dL vs. 84.7 ± 37.1 mg/dL; *P* = 0.013) compared with those without. FH increased the incidence of CVEs in patients with ACS [hazard ratio (HR): 3.058; 95% confidence interval (CI): 1.585–5.900; log-rank *P* < 0.001].

**Conclusion:**

FH is associated with an increased risk of CVEs in ACS and is an independent risk factor for ACS. This study highlights the importance of genetic testing of FH-related gene mutations in patients with ACS.

## Introduction

Familial hypercholesterolemia (FH) is a common genetic disorder that causes a remarkable increase in serum low-density lipoprotein cholesterol (LDL-C) levels, and results in xanthoma in the skin, subcutis, or tendon sheaths (1). FH can be divided into heterozygote FH, homozygote FH, compound heterozygote, and double heterozygote. Among these variants, heterozygote FH is more common. The prevalence of homozygote FH is estimated to be 1–3 in 1,000,000 in the general population, whereas that of heterozygote FH is higher, reaching 1 in 500 to 1 in 200 (1, 2). In China, adults with persistent serum LDL-C ≥ 135.3 mg/dL (3.5 mmol/L) without an apparent secondary cause of hypercholesterolemia should be screened for FH. The cutoff points are much lower than that followed by Western countries (3).

Elevated LDL-C level is a well-established factor that increases the risk of acute coronary syndrome (ACS) (4). The incidence of ACS in patients with untreated FH is 15–20 times higher than that in patients without FH (5, 6). Early identification of patients with FH can be helpful in adjusting their treatment regimen; moreover, the incidence of ACS can be reduced if appropriate treatment is administered before ACS onset. Therefore, it is important to accurately diagnose and screen FH in patients with ACS. However, the incidence of FH varies considerably in different regions owing to dietary and genetic factors. Among patients with ACS, the prevalence of FH was more than 5% in Europe (7, 8) and 2.7% in Japan (9).

Genetic testing is the gold standard for FH that identifies the presence of heterozygous pathogenic mutations in the genes associated with LDL-C metabolism. When combined with clinical features, next-generation sequencing can show high specificity and sensitivity in the identification of FH (10). However, genetic testing is rarely used to detect FH in patients with ACS owing to high economic costs among other reasons (1, 11, 12).

In this study, we evaluated the prevalence of molecularly defined FH in Chinese patients with ACS having LDL-C ≥ 135.3 mg/dL (3.5 mmol/L) and the effects of mutation on the incidence of cardiovascular events (CVEs) in a prospective observational cohort.

## Materials and methods

### Study population

This study was registered in the Chinese Clinical Trial Registry (identifier number: ChiCTR-EOC-17011463). Patients with ACS [ST-segment elevation myocardial infarction (STEMI), non-ST segment elevation myocardial infarction (NSTEMI), and unstable angina] having LDL-C levels ≥ 135.3 mg/dL (95th percentile of LDL-C among Chinese), and those > 18 years were consecutively recruited from March 1, 2017, to February 19, 2019, in the coronary care unit (CCU) of Union Hospital, Wuhan, China. The study was reviewed and approved by the ethical review board (Union Hospital, Tongji Medical College, Huazhong University of Science and Technology, China). Written informed consent was obtained from all participants enrolled in this study.

### DNA sequencing

Blood samples from patients were collected for DNA analysis. Genomic DNA samples were extracted, amplified, and sequenced following a previously reported procedure (13). The inactivating variants in genes with mutations known to cause FH (*LDLR*, *APOB*, *PCSK9*, and *LDLRAP1*), myopathy-associated variant in the *SLCO1B1* gene (rs4149056, rs4363657, and rs2306283), and genes functioning within the cholesterol-metabolism pathways (*APOE*, *CETP*, *LIPA*, LPL, and STAP1) were identified using a commercial FH Multigene Panel (Lipopro-1, Aegicare, China) (14). An average of 9.85 M (± 1.13 M) reads with 150 base paired end-sequencing strategy was obtained for the designed FH panel over all samples. The mean depth over targeted regions was 11628X (± 1215X), offering a high coverage of the regions of our interest. The mean coverage of selected target gene regions is shown in Supplementary Figure 1.

### Variant identification and genetic diagnosis of familial hypercholesterolemia

To identify FH-associated single nucleotide variants (SNVs) and Indels (short insertions and/or deletions of nucleotides < 50 bp), sequencing data were analyzed using a previously described in-house pipeline (13). Briefly, raw FASTQ files were processed with FASTP^1^ to detect and trim adapters and exclude low-quality reads. Clean reads were mapped to the GRCh37/hg19 reference genome using the Burrows-Wheeler Aligner (15). SNPs/Indels were identified using the Haplotype Caller tool of GATK and annotated using ANNOVAR (16). Annotation included genomic coordinates, functional role on protein coding, minor allele frequency, dbSNP identifiers, ClinVar identifiers, and deleteriousness-prediction scores of several prediction tools for pathogenicity of the variant (17). On average, 182 variants (± 15) were called for each sample, consisting of 154 SNPs (± 13) and 28 Indels (± 2).

A CNV kit was used to determine CNV in the LDLR gene (18). Normal references used for CNV identification were constructed using sequencing data from healthy subjects over the same DNA-sequencing protocol. During reference creation, the circular binary segmentation algorithm was chosen for CNV event segmentation, and the threshold parameter for copy-number calling was set to “−1.6, −0.8, 0.5, 1.” A previously published procedure was followed for process strategies that included normal reference creation, CNV event segmentation, and copy number reverting (13), except for the “target amplicon sequencing” mode, which was chosen to exclude off-target regions from calculation and disable edge correction. The detected CNVs were annotated using AnnotSV (19).

The identified variants were classified as pathogenic (P), likely pathogenic (LP), variants of uncertain significance (VUS), likely benign, or benign, following the ACMG criteria 8. Molecularly defined FH was defined as carrying the P or LP variants.

### Follow-up and outcome

The deadline for follow-up in this study was December 31, 2019. Follow-up details are described in Supplementary Material. CVEs were defined as cardiovascular death, non-fatal myocardial infarction, unstable angina pectoris requiring admission, unplanned coronary revascularization (unplanned percutaneous coronary intervention or coronary artery bypass graft driven by coronary ischemia), or heart failure requiring admission after discharge from the CCU.

The exclusion criteria, clinical measurements, and follow-up procedures are detailed in Supplementary Material.

### Statistical analysis

Baseline characteristic variables of continuous data are presented as mean ± standard deviation (SD), or median and interquartile range (IQR), as appropriate. Categorical variables are presented as absolute numbers (N) and percentages. Student’s *t*-test, one-way analysis of variance (ANOVA), or non-parametric tests were used to compare differences between groups of continuous parameters, as appropriate. Categorical variables were compared using the Chi-square test or Fisher’s exact test. A Kaplan-Meier curve with log-rank test was used to assess the percent of free time from CVEs. The prevalence of molecularly defined FH among patients with ACS and its corresponding 95% confidence interval (CI) was determined using normal approximation methods. Missing data in the lipid value at the last visit were replaced using the “last observation carried forward” method. Cox proportional hazards model was used to explore the relationship between FH and CVEs. Adjusted hazard ratio (HR) with 95% CI was used to measure the strength of association between CVEs and potential predictors. Statistical analyses were performed using R statistics version 4.0.0 (Arbor Day) and IBM SPSS Statistics version 22.0 (IBM SPSS Statistics, IBM Corporation). Statistical significance was defined using two-sided *P* < 0.05.

## Results

### Baseline clinical and genetic characteristics

**FIGURE 1:**
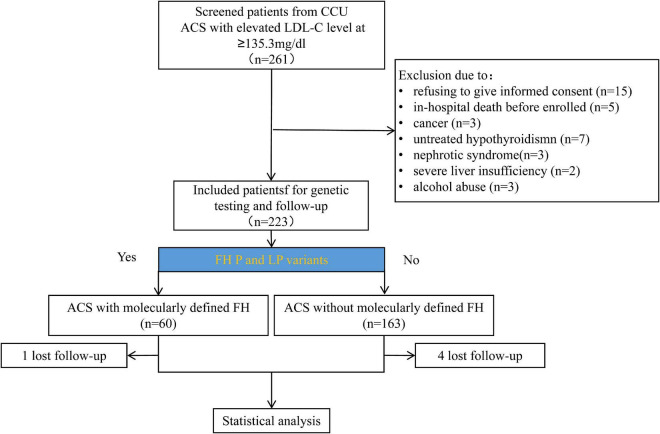
Flowchart of patient enrollment and follow-up.

A total of 261 patients hospitalized with ACS with elevated LDL-C levels of ≥ 135.3 mg/dL (Figure 1) were identified. Of these, 39 patients were excluded for the following reasons: rejection to participate (*n* = 15), in-hospital death before enrollment (*n* = 5), cancer (*n* = 3), untreated hypothyroidism (*n* = 7), nephrotic syndrome (*n* = 3), severe liver insufficiency (*n* = 2), and alcohol abuse (*n* = 3). Lastly, 223 patients were subjected to genetic testing and data analysis. CVE onset led to 51 patients ending the follow-up before December 31, 2019. There were 167 patients who ended follow-up on December 31, 2019. Five patients were lost to follow-up before the last scheduled visit of the study, and the last follow-up time was considered the end of the follow-up time.

Baseline demographics and clinical characteristics of enrolled patients with or without molecularly defined FH are shown in Table 1. All patients were of Asian (Chinese) descent. A total of 56.5% of study participants had a history of hypertension, 28.3% had a history of diabetes mellitus, 2.2% had a history of stroke, 38.1% were current smokers, and 45.7% had peripheral arteriosclerosis. In the sample, 24.7% of patients reported having previous coronary artery disease (CAD), and 13 patients (5.8%) were found to have xanthoma on physical examination. A total of 72.2% of study participants had angina pectoris and the remaining had acute myocardial infarction (AMI).

**TABLE 1:** Clinical characteristics of enrolled patients.

	Total (*n* = 223)	FH (*n* = 60)	Non-FH (*n* = 163)	*P*-value
Mean age at admission (years)	57.0 (50.0–65.0)	56.0 (46.0–66.0)	59.0 (52.0–65.0)	0.189
Female- n (%)	93 (41.7)	20 (33.3)	73 (44.8)	0.129
BMI	24.44 (22.27–26.50)	24.41 (22.14–26.28)	24.44 (22.41–26.54)	0.692
Hypertension- n (%)	126 (56.5)	29 (48.3)	97 (59.5)	0.170
Diabetes- n (%)	63 (28.3)	16 (26.7)	47 (28.8)	0.867
Previous Stroke- n (%)	5 (2.2)	2 (3.3)	3 (1.8)	0.613
Smoking- n (%)	85 (38.1)	23 (38.3)	62 (38.0)	1.000
Peripheral arteriosclerosis -n (%)	102 (45.7)	23 (38.3)	79 (48.5)	0.225
Previous CAD -n (%)	55 (24.7)	27 (45.0)	28 (17.2)	<0.001
Genesini score	42.0 (15.0–78.0)	66.0 (45.0–95.0)	28.0 (13.0–68.0)	<0.001
Xanthoma -n (%)	13 (5.8)	5 (8.3)	8 (4.9)	0.343
**CAD category**				
Unstable angina- n (%)	161 (72.2)	33 (55.0)	128 (78.5)	0.001
AMI- n (%)	62 (27.9)	27 (41.5)	35 (22.3)	0.001
Non-STEMI- n (%)	27 (12.1)	12 (20.0)	15 (9.2)	0.037
STEMI- n (%)	35 (15.7)	15 (25.0)	20 (12.3)	0.024
Revascularization treatment following baseline CAG -n (%)	120 (53.8)	39 (65.0)	81 (49.7)	0.049
PCI -n (%)	102 (44.6)	31 (47.7)	71 (45.2)	0.293
CABG -n (%)	18 (8.1)	8 (13.3)	10 (6.1)	0.097
On statins at enrollment -n (%)	59 (26.5)	26 (43.3)	33 (20.2)	0.001
**Data for biochemical research**				
hsTNI -ng/L	7.60 (2.20–138.30)	18.40 (4.32–282.22)	5.70 (1.80–63.25)	0.014
Total cholesterol - mg/dL	256.4 (240.1–284.6)	281.1 (246.3–316.7)	251.4 (239.0–275.7)	<0.001
Triglycerides - mg/dL	147.0 (110.7–208.1)	134.6 (101.7–209.9)	148.8 (110.7–206.4)	0.484
HDL-cholesterol - mg/dL	44.1 (37.5–53.8)	39.1 (33.3–50.7)	45.2 (39.1–54.5)	0.002
LDL-cholesterol - mg/dL	182.5 (167.8–208.4)	212.7 (175.9–233.6)	179.0 (166.7–194.9)	<0.001
non-HDL-C - mg/dL	209.2 (193.7–236.7)	235.5 (203.4–276.9)	204.6 (190.6–227.8)	<0.001

Of the 223 patients in the study, FH was molecularly diagnosed in 60 (26.9%) patients, who carried 61 P and LP variants (Supplementary Table 1). Fifty-nine patients (26.5%) were monoallelic FH variant carriers (57 heterozygous *LDLR* variant carriers, 2 heterozygous *APOB* variant carriers) and one patient was a biallelic FH variant carrier (compound heterozygous for *LDLR* variants). The 61 P/LP variants included 34 missenses, 9 non-senses, 3 frameshift indels, 12 splicing variants, and 3 CNVs (Supplementary Table 1). Twenty-two VUS were detected in 22 patients (Supplementary Table 2).

In general, there were no significant differences between the two groups with respect to age, gender, body mass index (BMI), prevalence of smoking; history of hypertension, diabetes mellitus, and stroke; and the incidence of peripheral arteriosclerosis or xanthoma. Notably, the prevalence of a prior history of CAD was much higher in patients with FH than in those without (45.0% vs. 17.2%; *P* < 0.001) (Table 1). The proportion of AMI was significantly higher in patients with FH compared with those without (41.5% vs. 22.3%; *P* = 0.001). The Gensini score revealed that coronary artery lesions in patients with FH were more severe than in those without FH (Gensini score 66.0 vs. 28.0, respectively; *P* < 0.001). Moreover, there were no significant differences in revascularization treatment among patients with and without FH. All patients received a statin alone and 106 (47.5%) received a statin/ezetimibe combination on discharge from the hospital. There were no significant differences in the rate of statin or ezetimibe treatment between the two groups (statins: 100% vs.100%, *P* = 1.000; ezetimibe: 55% vs. 44.8%, *P* = 0.226) (Table 2).

**TABLE 2:** Follow-up data of patients with or without FH.

	Total (*n* = 223)	FH (*n* = 60)	Non-FH (*n* = 163)	*P*-value
Follow-up time (months)	16.6 ± 7.1	14.5 ± 8.1	17.4 ± 6.5	0.014
CVEs -n (%)	51 (22.9)	24 (40.0)	27 (16.6)	<0.001
Non-fatal myocardial infarction	4	1	3	
Stroke	2	1	1	
Angina pectoris	30	13	17	
Heart failure	5	4	1	
Cardiovascular death	7	4	3	
Revascularization	2	0	2	
In-stent restenosis	1	1	0	
**Lipid-lowering drugs at discharge -n (%)**				
Statin	223 (100)	60 (100)	163 (100)	
Ezetimibe	106 (47.5)	33 (55)	73 (44.8)	0.226
**Lipid-lowering drugs at last visit -n (%)**				
Statin	122 (54.7)	29 (48.3)	93 (57.1)	0.289
Ezetimibe	19 (8.5)	7 (11.7)	12 (7.4)	0.416
**Lipid value at last visit**				
TC -mg/dL	138.1 ± 35.2	146.6 ± 44.5	135.0 ± 30.5	0.030
LDL-C -mg/dL	76.6 ± 29.8	84.7 ± 37.1	73.5 ± 25.9	0.013
**LDL-C value at last visit -n (%)**				
LDL-C ≥ 100 mg/dL	34 (15.2)	12 (20.0)	22 (8.6)	0.544
70 mg/dL ≤ LDLC-C < 100 mg/dL	83 (37.2)	26 (43.3)	57 (35.0)	0.276
LDL-C < 70 mg/dL	104 (46.6)	21 (35.0)	83 (50.9)	0.049
LDL-C reduction	117.6 ± 42.9	134.6 ± 67.3	111.8 ± 27.5	<0.001
LDL-C reduction rate	60.7% (95% CI 58.9–62.4)	61.3% (95% CI 57.7–64.8)	60.4% (95% CI 58.5–62.4)	0.677

The baseline lipid profiles of all patients were obtained before commencing lipid-lowering therapy (Table 1). The presence of pathogenic FH in patients resulted in significantly higher levels of both total cholesterol (TC) and LDL-C compared with those without FH (TC: 281.1 mg/dL vs. 251.4 mg/dL, *P* < 0.001; LDL: 212.7 mg/dL vs. 179.0 mg/dL, *P* < 0.001), but a decrease in HDL-C levels (39.1 mg/dL vs. 45.2 mg/dL; *P* = 0.002). There were no significant differences in triglyceride levels (134.6 mg/dL vs. 148.8 mg/dL; *P* = 0.484) between the groups.

### Changes in lipid profiles among patients with acute coronary syndrome with or without familial hypercholesterolemia

Changes in lipid profiles in the laboratory results and the statin drug treatment during the out-of-hospital follow-up are reported in Table 2. During follow-up, the missing data from analyzed patients was < 10%. LDL-C levels were available at follow-up for all 223 patients. The mean TC and LDL-C levels at the last visit were 138 and 76.6 mg/dL, respectively. At the last visit, 34 patients (15.2%) had LDL-C levels ≥ 100 mg/dL, 83 patients (37.2%) had LDL-C levels between 70 and 100 mg/dL, and 104 patients (46.6%) had LDL-C levels < 100 mg/dL (Table 2).

Although there were no significant differences between discharged patients with and without FH among those receiving statin treatment, the FH pathogenic variants led to an increase in median LDL-C at the last visit (84.7 mg/dL vs. 73.5 mg/dL; *P* = 0.013) and an absolute reduction in LDL-C (134.6 mg/dL vs. 111.8 mg/dL; *P* < 0.001). There was no significant difference in the proportion of decline (60.4% vs. 61.3%; *P* = 0.677) between the two groups (Table 2). Moreover, the percentage of patients with FH having LDL-C levels < 70 mg/dL was slightly lower than those without FH; however, there were no differences between the two groups in the proportion of patients with LDL-C levels between 70 and 100 mg/dL or LDL-C levels ≥ 100 mg/dL at the last visit.

### Prognosis of patients with acute coronary syndrome based on familial hypercholesterolemia status

During the follow-up period (16.6 ± 7.1 months), 51 (22.9%) patients developed CVEs. Seven patients (3.1%) died (four patients with FH), four (1.8%) had a non-fatal myocardial infarction, 30 (13.5%) experienced unstable angina pectoris requiring admission, two (0.9%) received unplanned revascularization, and five (2.2%) experienced heart failure. Moreover, there was no difference in the proportion of patients receiving statin drug treatment during the follow-up period between those with and without FH.

**FIGURE 2:**
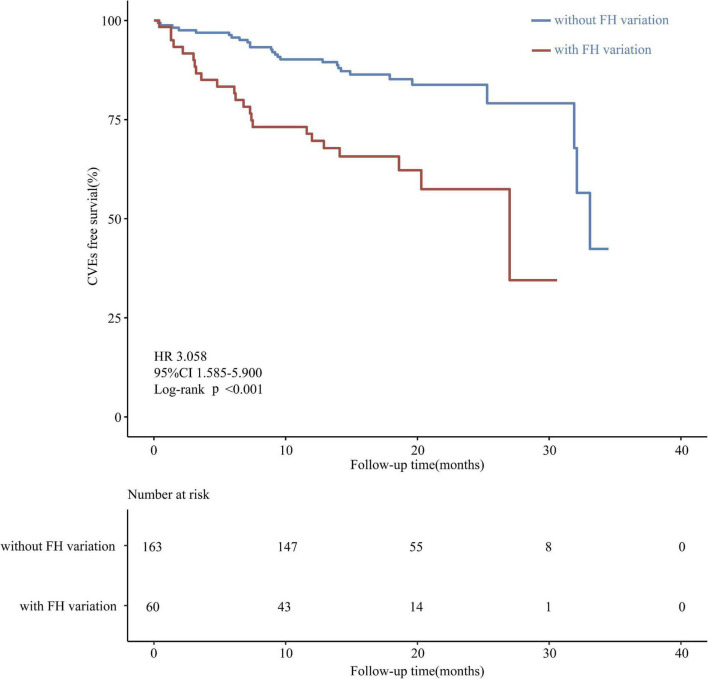
Kaplan-Meier curve of the cumulative event-free survival analyzed according to variant classification based on the ACMG/AMP criteria.

**FIGURE 3:**
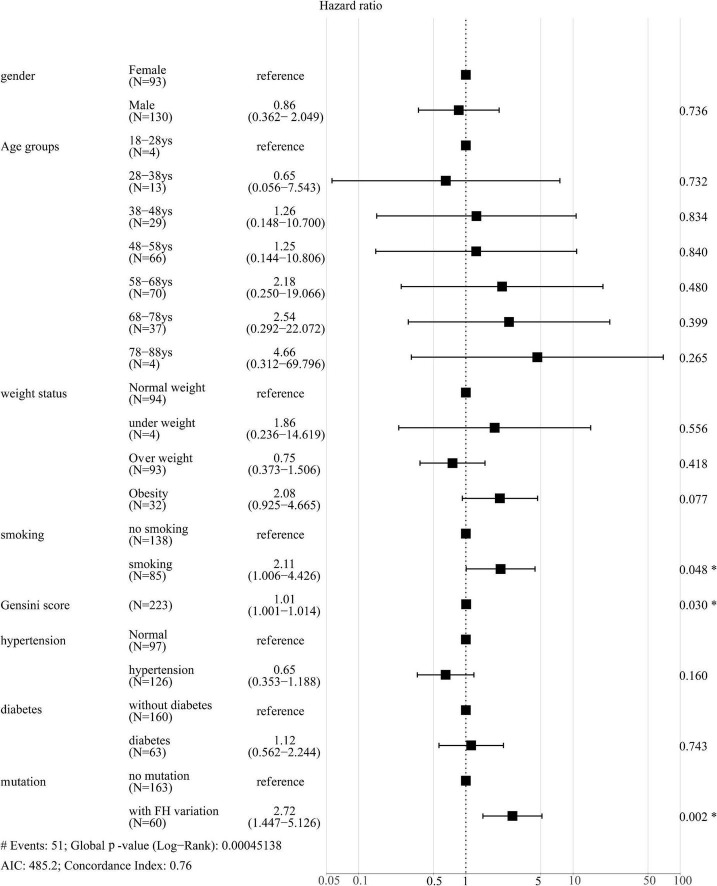
Forest plot of hazard ratios using multivariate Cox regression analysis of the effect of different parameters on CVEs. *P < 0.05.

Next, the correlation between pathogenic variants and risk factors with CVEs was analyzed. Among the 51 CVEs, 24 and 27 CVEs were observed in patients with and without FH, respectively. Kaplan-Meier curves revealed that patients with the FH P/LP variants had a much higher incidence of CVEs (HR: 3.058; 95% CI: 1.585–5.900 log-rank *P* < 0.001) (Figure 2). As the carrier with VUS was classified as the non-FH group, which could affect the relationship between FH P/LP variants and the risk of CVEs, Kaplan-Meier analysis was performed when patients with VUS were classified in the FH group or excluded (Supplementary Figure 2). The incidence of CVEs was significantly higher in patients with the FH P/LP variants than those with the FH likely benign/benign variants (HR: 2.717; 95% CI: 1.437–5.138, log-rank *P* < 0.001) (Supplementary Figure 2A), and the incidence of CVEs was also much higher in patients with the FH P/LP/VUS variants than in those with the FH likely benign/benign variants (HR: 1.917; 95% CI: 1.071–3.431, log-rank *P* = 0.016) (Supplementary Figure 2B). These findings confirmed the robustness of the results with VUS in patients with FH. The risk of CVD is associated with gender, age, BMI, Gensini score, history of smoking, hypertension, and diabetes. Multivariate Cox regression analysis was used to estimate these risk factors. The forest map shows that the incidence of CVEs is not significantly different between patients of different genders, ages, and BMI. Moreover, a history of hypertension and diabetes did not affect the rate of CVEs in this study population (Figure 3), whereas a history of smoking promoted the rate of CVEs in patients with ACS (HR: 2.11; 95% CI: 1.006–4.426, *P* = 0.048) (Figure 3). Moreover, the results showed that a high Gensini score was associated with higher CVE rates (HR: 1.01; 95% CI: 1.001–1.014, *P* = 0.030) (Figure 3). Moreover, patients with FH P/LP variants had an increased risk of CVEs (HR: 2.72; 95% CI: 1.447–5.126, *P* = 0.002) (Figure 3), independent of these factors.

## Discussion

In this study, we have described a genetic analysis of FH in suspected ACS patients with LDL-C levels ≥ 135.3 mg/dL. The prevalence of molecularly defined FH in these patients was 26.9%. Coronary artery lesions in patients with FH were more severe than in those without FH. After a follow-up of 16.6 months, on average, we found that the FH pathogenic variants caused an increase in the incidence of CVEs.

A previous study has reported a considerably low prevalence of FH in China (20). This low detection rate of FH diagnosis is attributable to the lack of emphasis on genetic screening in the general population, especially in many developing countries. We observed that 26.9% of patients with ACS having LDL-C levels ≥ 135.3 mg/dL carried FH-related mutations, which was also much higher than that reported previously in Western countries and Japan (5, 21, 22). It is well known that adults hospitalized for ACS have a higher prevalence of FH than the general population (7). Besides, three CNV carriers were found in our study, which also improved the detection rate of FH. The above differences may be attributed to different nationalities and the lower average level of cholesterol in the Chinese population.

With changes in the nutrient composition and an increased intake of high-calorie foods, the number of patients with hypercholesterolemia identified based on physical examination has rapidly increased in China. The discovery of a large number of patients with ACS with hypercholesterolemia suggests that FH is a common disease and that its diagnosis and treatment in China are not adequate. Thus, it represents a global public health burden as it is a major cause of early CAD (1). Early detection of FH can lead to targeted dietary control and more effective treatment program with pharmacological agents. Expanded genetic screening and cascade testing have also been recommended.

As the prevalence of FH is significantly higher in patients with ACS than in the general population, FH gene testing is necessary for patients with ACS. Previous clinical studies on lipid profiles have mainly assessed the prevalence and cardiovascular prognosis of patients with FH as suggested by the Simon Broome (SB) algorithms in the United Kingdom and the Dutch Lipid Clinic (DLC) algorithm, and not by genetic screening. However, these clinical algorithms do not apply to children for the diagnosis of FH, as the clinical DLC and SB algorithms may be not valid in these populations and in subjects with normal LDL-C levels in the absence of molecular genetic testing, which may lead to the inaccurate diagnosis of FH (23–26). Genetic tests can provide a more accurate molecular diagnosis for patients with FH; thus, these tests should be considered the standard diagnostic procedure for patients with possible FH, which could help reduce the incidence of ACS (27).

The findings from our study suggest that FH increases the risk of CVEs after multivariate adjustment for common risk factors. Moreover, we found that the pathogenic variants of FH led to an increase in LDL-C levels even after treatment with lipid-lowering drugs (statins combined with ezetimibe). These findings indicate that knowledge on the FH pathogenic variation status can provide information on the additional risks, apart from the clinical data, in identifying individuals with ACS who are at the highest risk. Accordingly, these patients should be instructed to initiate early lipid-lowering therapy to improve the clinical outcomes of ACS. In patients with ACS with pathogenic FH, more intensive lipid-lowering therapy (PCSK9 inhibitors, higher-intensity statins) may be necessary to further reduce LDL-C levels.

An increasing number of lipid-lowering treatments, such as PCSK9 inhibitors and lipid adsorption therapy are available to treat FH. Moreover, the novel gene-editing CRISPR technology could actually change these gene-driven diseases (28, 29). The intake of dietary supplements and functional foods and performance of physical activities are also considered beneficial in maintaining a normal lipid profile (30, 31).

## Conclusion

We found that FH was present in 26.9% of patients in China with ACS having LDL-C ≥ 135.3 mg/dL, which may be a more beneficial approach compared with earlier genetic screening. FH-associated pathogenic variants can provide the prognostic evidence for ACS and risk-stratification information that is independent of LDL-C levels. Early recognition of FH by genetic testing can help change the treatment strategy for patients with ACS and accordingly alter their prognosis.

## Limitations

Our study has several limitations. First, this is a single-center, prospective cohort study with a small sample size, where only a few CVEs were observed. Further in-depth studies including multicenter clinical studies are, therefore, needed to confirm our study findings. Second, the sequencing panel used in our study only contained eight most common genes and may have been missing the polygenic FH gene and other genes related to lipid metabolism. The majority of mutations were found in the LDLR gene. Third, as the clinical data in this study were obtained before PCSK9 inhibitors were marketed in China, patients in this study were not treated with PCSK9 inhibitors. Fourth, the original planned follow-up time of this study was 3 years. Due to the onset of the COVID-19 pandemic in Wuhan, the follow-up study could not be conducted. As positive results were observed in this study, it was terminated early. Lastly, unknown confounding factors may exist, although multiple confounding factors were adjusted for in the Cox regression analysis.

## Data availability statement

The raw data reported in this article are deposited in https://doi.org/10.6084/m9.figshare.20304711.v1.

## Ethics statement

The studies involving human participants were reviewed and approved by the Ethics Committee of Wuhan Union Hospital in China. The patients/participants provided their written informed consent to participate in this study.

## Author contributions

FXZ and FZ conceived and designed the study. CW, FXZ, and FZ wrote the manuscript. All authors performed the experiments, analyzed the data, contributed to the article, and approved the submitted version.

## Conflict of interest

The authors declare that the research was conducted in the absence of any commercial or financial relationships that could be construed as a potential conflict of interest.

## Publisher’s note

All claims expressed in this article are solely those of the authors and do not necessarily represent those of their affiliated organizations, or those of the publisher, the editors and the reviewers. Any product that may be evaluated in this article, or claim that may be made by its manufacturer, is not guaranteed or endorsed by the publisher.

## Footnotes

1
https://github.com/OpenGene/fastp

## Abbreviations

FH: Familial hypercholesterolemia
ACS: Acute coronary syndrome
CVE: Cardiovascular event
STEMI: ST-segment elevation myocardial infarction
NSTEMI: Non-ST segment elevation myocardial infarction
CCU: Coronary care unit
AMI: Acute myocardial infarction
CNV: Copy number variation
TC: Total cholesterol
LDL-C: Low-density lipoprotein cholesterol
PCSK9: Proprotein convertase subtilisin/kexin type 9
CAD: Coronary artery disease
VUS: Variant of uncertain significance
LP: Likely pathogenic
ACMG: American College of Medical Genetics and Genomics
AMP: The Association for Molecular Pathology.
