# Supplementary material for: Prevalence and prognosis of molecularly defined familial hypercholesterolemia in patients with acute coronary syndrome
Source: Front Cardiovasc Med. 2022 Jul 27;9:921803. doi: 10.3389/fcvm.2022.921803 (PMC9363594; doi:10.3389/fcvm.2022.921803)
Supplement: Supplementary file 1 [file Data_Sheet_1.doc]

**Supplementary methods**

**Exclusion criteria**

Patients were excluded if they were unwilling, had a significant hematologic disorder on admission, were pregnant, had cancer, or had a secondary cause of hypercholesterolemia (e.g., untreated hypothyroidism, nephrotic syndrome, severe liver insufficiency, and alcohol abuse).

**Clinical measurements**

Demographic and clinical characteristics of patients were obtained from electronic medical records, and the information was supplemented by questionnaires. During admission, fasting blood samples were obtained to measure LDL-C levels, which were corrected using Friedewald formula if patients were already on statin therapy ^1^. Blood pressure was measured in the supine position. All patients underwent coronary arteriography during hospitalization, and the Gensini score was used to score the severity of coronary artery disease (CAD).

**Follow-up detail**

All included patients were followed up during clinical visits every 6 months and interviewed in-person at the clinic by well-trained cardiologists (patients who could not reach the clinic on time was followed up through a telephonic chat or the WeChat app), who were blinded to the results of genetic testing until the CVEs occurred or up to the last day of the follow-up period. Aspirin and clopidogrel or ticagrelor were used for all patients. Clinical data and CVEs were recorded in a web-based data system.

**Supplementary Figure legends**

**Supplementary Figure 1.**

Representative examples of the sequence enrichment of *LDLR*, *APOB*, *PCSK9* and *LDLRAP1* genes. Exons of the genes are shown as RefSeq annotation tracks.

**Supplementary Figure 2.**

Kaplan-Meiers curve of the cumulative event-free survival analyses according to variants classification of the ACMG/AMP criteria. (a) incidence of CVEs in patients with the FH P/LP variants and patients with the FH likely benign/benign variants; (b) incidence of CVEs in patients with the FH P/LP/VUS variants and patients with the FH likely benign/benign variants. P=pathogenic, LP=likely pathogenic, VUS=variant of uncertain clinical significance.

**Supplementary Table 1. Profiles of “pathogenic” or “likely pathogenic” variants according to the American College of Medical Genetics and Genomics (ACMG) guidelines in FH patients with ACS.**

| **Patient ID** | **Gene** | **Transcript** | **cDNA change** | **AA change** | **Types of variation** | **gnomAD All_MAF** | **ACMG classification** | **ClinVar Accession** |
| --- | --- | --- | --- | --- | --- | --- | --- | --- |
| **FH_1746** | *LDLR* | NM_000527 | c.G986A | p.C329Y | Missense | 0.00009687 | Likely Pathogenic | VCV000226344 |
| **AS1167** | *LDLR* | NM_000527 | c.940+1G>A | - | Splicing | NA | Pathogenic | VCV000251541 |
| **FH_1765** | *LDLR* | NM_000527 | c.C939A | p.C313X | Nonsense | NA | Pathogenic | VCV000251539 |
| **19L0000876** | *LDLR* | NM_000527 | c.C939A | p.C313X | Nonsense | NA | Pathogenic | VCV000251539 |
| **19L0000842** | *LDLR* | NM_000527 | c.T691C | p.C231R | Missense | NA | Likely Pathogenic | VCV000251396 |
| **AS2756** | *LDLR* | NM_000527 | c.T691C | p.C231R | Missense | NA | Likely Pathogenic | VCV000251396 |
| **18L0002014** | *LDLR* | NM_000527 | c.G682T | p.E228X | Nonsense | 0.00003231 | Pathogenic | VCV000226333 |
| **19L0000326** | *LDLR* | NM_000527 | c.G665T | p.C222F | Missense | NA | Likely Pathogenic | VCV000251362 |
| **18L0000303** | *LDLR* | NM_000527 | c.656_657delG | p.Gly304AlafsTer46 | Frameshift | NA | Pathogenic | Novel |
| **18L0000394** | *LDLR* | NM_000527 | c.G622T | p.E208X | Nonsense | NA | Pathogenic | VCV000251329 |
| **18L0002175** | *LDLR* | NM_000527 | c.G622A | p.E208K | Missense | NA | Likely Pathogenic | VCV000251328 |
| **18L0002829** | *LDLR* | NM_000527 | c.2312-156_*116del | - | CNV | NA | Pathogenic | Novel |
| **18L0000334** | *LDLR* | NM_000527 | c.2390-93_2547+224del | - | CNV | NA | Pathogenic | Novel |
| **FH_2276** | *LDLR* | NM_000527 | c.C621T | - | Splicing | NA | Pathogenic | VCV000003747.3 |
| **18L0000242** | *LDLR* | NM_000527 | c.C621T | - | Splicing | NA | Pathogenic | VCV000003747.3 |
| **18L0002063** | *LDLR* | NM_000527 | c.G502T | p.D168Y | Missense | NA | Likely Pathogenic | VCV000251259 |
| **19L0000346** | *LDLR* | NM_000527 | c.T482C | p.I161T | Missense | NA | Likely Pathogenic | VCV000440575 |
| **18L0002090** | *LDLR* | NM_000527 | c.G428A | p.C143Y | Missense | NA | Likely Pathogenic | VCV000251220 |
| **18L0000324** | *LDLR* | NM_000527 | c.G2413A | p.G805R | Missense | NA | Likely Pathogenic | VCV000252328 |
| **19L0000878** | *LDLR* | NM_000527 | c.A2344T | p.K782X | Nonsense | NA | Pathogenic | VCV000226392 |
| **20180706L001** | *LDLR* | NM_000527 | c.2096delC | p.Pro699ArgfsTer10 | Frameshift | NA | Pathogenic | Novel |
| **18L0000387** | *LDLR* | NM_000527 | c.C2054T | p.P685L | Missense | NA | Likely Pathogenic | VCV000003702.11 |
| **FH_1891** | *LDLR* | NM_000527 | c.C2054T | p.P685L | Missense | NA | Likely Pathogenic | VCV000003702.11 |
| **18L0001989** | *LDLR* | NM_000527 | c.G2026C | p.G676R | Missense | NA | Likely Pathogenic | VCV000252176.1 |
| **18L0002838** | *LDLR* | NM_000527 | c.2312-156_*116del | - | CNV | NA | Pathogenic | Novel |
| **FH_1881** | *LDLR* | NM_000527 | c.G2026C | p.G676R | Missense | NA | Likely Pathogenic | VCV000252176.1 |
| **18L0002178** | *LDLR* | NM_000527 | c.G2026C | p.G676R | Missense | NA | Likely Pathogenic | VCV000252176.1 |
| **19L0000834** | *LDLR* | NM_000527 | c.1987+1G>T | - | Splicing | NA | Pathogenic | Novel |
| **FH_1745** | *LDLR* | NM_000527 | c.191-1G>A | - | Splicing | NA | Pathogenic | VCV000251071 |
| **19L0000324** | *LDLR* | NM_000527 | c.C1880T | p.A627V | Missense | NA | Likely Pathogenic | VCV000252102 |
| **18L0002128** | *LDLR* | NM_000527 | c.G1879A | p.A627T | Missense | NA | Likely Pathogenic | VCV000252101 |
| **20180613L001** | *LDLR* | NM_000527 | c.G1879A | p.A627T | Missense | NA | Likely Pathogenic | VCV000252101 |
| **20181018L002** | *LDLR* | NM_000527 | c.G1879A | p.A627T | Missense | NA | Likely Pathogenic | VCV000252101 |
| **18L0002015** | *APOB* | NM_000384 | c.C10579T | p.R3527W | Missense | 0.0000646 | Likely Pathogenic | VCV000040223.14 |
| **18L0002858** | *LDLR* | NM_000527 | c.G1879A | p.A627T | Missense | NA | Likely Pathogenic | VCV000252101 |
| **AS1136** | *LDLR* | NM_000527 | c.G1879A | p.A627T | Missense | NA | Likely Pathogenic | VCV000252101 |
| **AS1169** | *LDLR* | NM_000527 | c.G1879A | p.A627T | Missense | NA | Likely Pathogenic | VCV000252101 |
| **AS1149** | *LDLR* | NM_000527 | c.G1879A | p.A627T | Missense | NA | Likely Pathogenic | VCV000252101 |
| **AS3318** | *LDLR* | NM_000527 | c.G1879A | p.A627T | Missense | NA | Likely Pathogenic | VCV000252101 |
| **18L0002120** | *LDLR* | NM_000527 | c.G1879A | p.A627T | Missense | NA | Likely Pathogenic | VCV000252101 |
| **18L0000397** | *LDLR* | NM_000527 | c.G1879A | p.A627T | Missense | NA | Likely Pathogenic | VCV000252101 |
| **FH_1760** | *LDLR* | NM_000527 | c.G1879A | p.A627T | Missense | NA | Likely Pathogenic | VCV000252101 |
| **18L0002051** | *LDLR* | NM_000527 | c.G1879A | p.A627T | Missense | NA | Likely Pathogenic | VCV000252101 |
| **AS1147** | *LDLR* | NM_000527 | c.G1774A | p.G592R | Missense | 0.000016 | Likely Pathogenic | VCV000373769.4 |
| **18L0000298** | *LDLR* | NM_000527 | c.1705+1G>A | - | Splicing | NA | Pathogenic | VCV000226367 |
| **FH_1923** | *LDLR* | NM_000527 | c.1567G>A | p.V523M | Missense | NA | Likely pathogenic​ | VCV000003696.14 |
| **FH_1553** | *LDLR* | NM_000527 | c.C1467G | p.Y489X | Nonsense | 0.000004 | Pathogenic | VCV000440647 |
| **18L0000238** | *LDLR* | NM_000527 | c.G1448A | p.W483X | Nonsense | NA | Pathogenic | VCV000226356.5 |
| **19L0000832** | *LDLR* | NM_000527 | c.G1448A | p.W483X | Nonsense | NA | Pathogenic | VCV000226356.5 |
| **18L0002929** | *LDLR* | NM_000527 | c.C1257A | p.Y419X | Nonsense | NA | Pathogenic | VCV000251758 |
| **18L0002929** | *LDLR* | NM_000527 | c.G1879A | p.A627T | Missense | NA | Likely pathogenic | VCV000252101 |
| **18L0000377** | *LDLR* | NM_000527 | c.G1247A | R416Q | Missense | 0.00003232 | Likely pathogenic | VCV000251752.6 |
| **18L0000379** | *LDLR* | NM_000527 | c.C1246T | R416W | Missense | NA | Likely pathogenic | VCV000183110.8 |
| **19L0000327** | *LDLR* | NM_000527 | c.C1216A | - | Splicing | NA | Pathogenic | VCV000003746.6 |
| **AS2811** | *LDLR* | NM_000527 | c.C1216A | - | Splicing | NA | Pathogenic | VCV000003746.6 |
| **FH_1896** | *LDLR* | NM_000527 | c.C1216A | - | Splicing | NA | Pathogenic | VCV000003746.6 |
| **18L0002007** | *LDLR* | NM_000527 | c.C1216A | - | Splicing | NA | Pathogenic | VCV000003746.6 |
| **19L0000296** | *LDLR* | NM_000527 | c.1187-10G>A | - | Splicing | NA | Pathogenic | VCV000226349.7 |
| **18L0002857** | *LDLR* | NM_000527 | c.1187-10G>A | - | Splicing | NA | Pathogenic | VCV000226349.7 |
| **18L0000399** | *LDLR* | NM_000527 | c.T2G | p.M1R | Missense | NA | Likely pathogenic | Novel |
| **18L0000248** | *APOB* | NM_000384 | c.3565_3566delT | p.Met1189ArgfsTer33 | frameshift | NA | Pathogenic | Novel |

**Supplementary Table 2. Profiles of “variants of uncertain significance (VUS)” according to the American College of Medical Genetics and Genomics (ACMG) guidelines in FH patients with ACS.**

| **Patient ID** | **Gene** | **Transcript** | **cDNA change** | **AA change** | **Types of variation** | **gnomAD All_MAF** | **ACMG classification** | **ClinVar Accession** |
| --- | --- | --- | --- | --- | --- | --- | --- | --- |
| **18L0000290** | *APOB* | NM_000384 | c.G215T | p.S72I | Missense | 0.00003229 | VUS | NA |
| **18L0002871** | *PCSK9* | NM_174936 | c.G103A | p.D35N | Missense | NA | VUS | Novel |
| **18L0000347** | *PCSK9* | NM_174936 | c.G658A | p.A220T | Missense | 0.00003231 | VUS | VCV000440718 |
| **18L0002010** | *APOB* | NM_000384 | c.G1342A | p.A448T | Missense | NA | VUS | VCV000630352 |
| **FH_1854** | *APOB* | NM_000384 | c.C7757T | p.T2586I | Missense | NA | VUS | Novel |
| **18L0001974** | *APOB* | NM_000384 | c.G676A | p.A226T | Missense | 0.00003228 | VUS | VCV000630867 |
| **18L0001995** | *APOB* | NM_000384 | c.C5828T | p.S1943F | Missense | NA | VUS | Novel |
| **18L0002106** | *PCSK9* | NM_174936 | c.G658A | p.A220T | Missense | 0.00003231 | VUS | VCV000630867 |
| **18L0000317** | *STAP1* | NM_001317769 | c.T5C | p.M2T | Missense | 0.0008 | VUS | NA |
| **TU1264** | *LDLR* | NM_000527 | c.268G>A | p.D90N | Missense | 0.00009688 | VUS | VCV000251105 |
| **18L0000343** | *APOB* | NM_000384 | c.A3077G | p.D1026G | Missense | NA | VUS | Novel |
| **18L0000367** | *LDLR* | NM_000527 | c.G268A | p.D90N | Missense | 0.00009688 | VUS | VCV000251105 |
| **18L0000367** | *LDLRAP1* | NM_015627 | c.G138A | p.M46I | Missense | NA | VUS | Novel |
| **FH_1917** | *APOB* | NM_000384 | c.G1342A | p.A448T | Missense | NA | VUS | VCV000630352 |
| **FH_1748** | *LDLR* | NM_000527 | c.C1243T | p.H415Y | Missense | NA | VUS | Novel |
| **18L0000380** | *PCSK9* | NM_174936 | c.G658A | p.A220T | Missense | NA | VUS | VCV000630867 |
| **18L0000390** | *LDLR* | NM_000527 | c.C1747T | p.H583Y | Missense | 0.00006459 | VUS | VCV000200921 |
| **18L0000333** | *APOB* | NM_000384 | c.C2395T | p.L799F | Missense | 0.00003229 | VUS | NA |
| **18L0002102** | *STAP1* | NM_001317769 | c.T5C | p.M2T | Missense | 0.0008 | VUS | NA |
| **18L0000252** | *LDLR* | NM_000527 | c.C1747T | p.H583Y | Missense | 0.00006459 | VUS | VCV000200921 |
| **AS1166** | *LDLR* | NM_000527 | c.C1747T | p.H583Y | Missense | 0.00006459 | VUS | VCV000200921 |
| **FH_1757** | *STAP1* | NM_001317769 | c.T5C | p.M2T | Missense | 0.0008 | VUS | NA |
| **18L0002922** | *LDLR* | NM_000527 | c.G728A | p.C243Y | Missense | NA | VUS | Novel |

**Reference**

1. Friedewald WT, Levy RI, Fredrickson DS. Estimation of the concentration of low-density lipoprotein cholesterol in plasma, without use of the preparative ultracentrifuge*. Clin Ch*em. 1972;18:499-502
